# Construction of a high-density, high-quality genetic map of cultivated lotus (*Nelumbo nucifera*) using next-generation sequencing

**DOI:** 10.1186/s12864-016-2781-4

**Published:** 2016-06-17

**Authors:** Zhengwei Liu, Honglian Zhu, Yuping Liu, Jing Kuang, Kai Zhou, Fan Liang, Zhenhua Liu, Depeng Wang, Weidong Ke

**Affiliations:** Institute of Vegetable, Wuhan Academy of Agriculture Science and Technology, Wuhan, Hubei 430065 China; Nextomics Biosciences Co., Ltd., Wuhan, Hubei China

**Keywords:** Double digest RADSeq, *Nelumbo nucifera*, Molecular breeding, Assembly anchoring, Single-nucleotide polymorphisms, Next generation sequencing, Genetic map

## Abstract

**Background:**

The sacred lotus (*Nelumbo nucifera*) is widely cultivated in China for its edible rhizomes and seeds. Traditional plant breeding methods have been used to breed cultivars with increased yields and quality of rhizomes and seeds with limited success. Currently, the available genetic maps and molecular markers in lotus are too limited to be useful for molecular genetics based breeding programs. However, the development of next-generation sequencing (NGS) technologies has enabled large-scale identification of single-nucleotide polymorphisms (SNPs) for genetic map construction. In this study, we constructed an SNP-based high-density genetic map for cultivated lotus using double digest restriction site-associated DNA sequencing (ddRADseq).

**Results:**

An F_2_ population of 96 individuals was derived from a cross between the rhizome lotus cultivar ‘Juwuba’ (male parent) and the seed lotus cultivar ‘Mantianxing’ (female parent). Genomic DNAs from this population were digested with the restriction enzymes EcoRI and MspI and then sequenced. In total, 133.65 Gb of raw data containing 1,088,935,610 pair-end reads were obtained. The coverage of reads on a reference genome was 7.2 % for the female parent, 6.56 % for the male parent, and 1.46 % for F_2_ individuals. From these reads, 10,753 valid SNP markers were used for genetic map construction. Finally, 791 bin markers (so-segregated adjacent SNPs treated as a bin marker), consisting of 8,971 SNP markers, were sorted into 8 linkage groups (LGs) that spanned 581.3 cM, with an average marker interval of 0.74 cM. A total of 809 genome sequence scaffolds, covering about 565.9 cM of the wild sacred lotus genome, were anchored on the genetic map, accounting for 70.6 % of the genome assembly.

**Conclusions:**

This study reports the large-scale discovery of SNPs between cultivars of rhizome and seed lotus using a ddRADseq library combined with NGS. These SNPs have been used to construct the first high-density genetic map for cultivated lotus that can serve as a genomic reference and will facilitate genetic mapping of important traits in the parental cultivars.

**Electronic supplementary material:**

The online version of this article (doi:10.1186/s12864-016-2781-4) contains supplementary material, which is available to authorized users.

## Background

*Nelumbo nucifera* Gaertn., also known as sacred lotus, is an aquatic crop of considerable agricultural, ornamental, religious, and medical importance [1, 2]. Sacred lotus belongs to the family Nelumbonaceae (2n = 16, genome size is about 929 Mb)*,* which comprises only two species, *N.nucifera* and *N.lutea* [3]. Analyses of morphological differences and molecular markers have identified three distinct types of cultivar: rhizome lotus, seed lotus, and flower lotus [4, 5]. Rhizome lotus and seed lotus are the two most popular aquatic vegetable crops in China [6] with estimated areas under cultivation of 660,000 and 67,000 ha, respectively, in 2012.

Lotus breeding began about 30 years ago in China [7] and several elite lotus cultivars have been produced and are now widely cultivated [8]. Nevertheless, research on lotus still faces many difficulties compared to other crop species as it is labor-intensive, time consuming, and costly. Firstly, single lotus plants can span three to five square meters and need to be grown in separate cement ponds to prevent misidentification of individuals, which means a high investment in the cultivation facility. Secondly, the underground rhizomes of lotus can expand to 30 to 50 cm in depth in the soil, which increases the difficulty of rhizome phenotyping. As a result of these complications, the genetic basis of the most important agronomic traits and other phenotypes are not yet understood.

Plant breeding using molecular genetic markers is an efficient approach to overcoming such limitations. High-density genetic maps, including quantitative trait locus (QTL) mapping and marker-assisted selection, are essential for the efficient use of this approach to plant breeding. Yang et al. [9] reported the first genetic maps for *N. nucifera* and *N. lutea* based on 47 and 177 SSR (simple sequence repeat) markers, respectively. These maps were later expanded by Zhang et al. [1, 10]. Currently, the *N.nucifera* genetic map comprises 224 markers and the *N. lutea* genetic map has 3895 RADseq markers and 156 SSR markers on 9 linkage groups (LGs). These genetic maps are of value for mapping quantitative trait loci (QTLs) affecting plant size, leaf shape, petal shape and color, and other desirable characters; however, they are less useful for mapping yield-related traits in rhizome and seed lotuses since the parental plants were wild lotus phenotypes that exhibit unfavorable agronomic traits such as thin rhizomes and low seed yield. In addition, the existing genetic map for *N. nucifera* also lacks sufficient markers to conduct QTL analysis and molecular mapping, while the high-density genetic map for *N. lutea* has failed to coalesce into eight linkage groups representing the eight lotus chromosomes. As a result, construction of a more saturated map with a higher density of markers is needed to meet the demand for breeding improved lotus cultivars especially for crop varieties.

Next-generation sequencing (NGS) provides the opportunity for large-scale genome sequencing, and has already been exploited in *denovo* sequencing of disparate organisms such as panda [11], cucumber [12], apple [13], and lotus [1, 2]. Ming et al. successfully sequenced the genome of the sacred lotus cultivar ‘China Antique’ [1], while a draft genome of a wild strain of lotus was reported that spanned 792 Mb, 85.2 % of the estimated 929 Mb lotus genome. Single-nucleotide polymorphisms (SNPs) are the most abundant and stable form of genetic variation in most genomes. Large-scale identification of high quality SNPs can be achieved through use of restriction site-associated DNA sequencing (RADseq); this method has the advantages of relatively low cost and speed [14], which are highly beneficial for genetic map construction. RADseq is based on sequencing the DNA flanking specific restriction enzyme sites rather than the whole genome [15]. The use of two restriction enzymes, double digest RADseq (ddRADseq), improves the efficiency of producing a sequencing library and robustness of the results while minimizing cost [16].

**Fig. 1:**
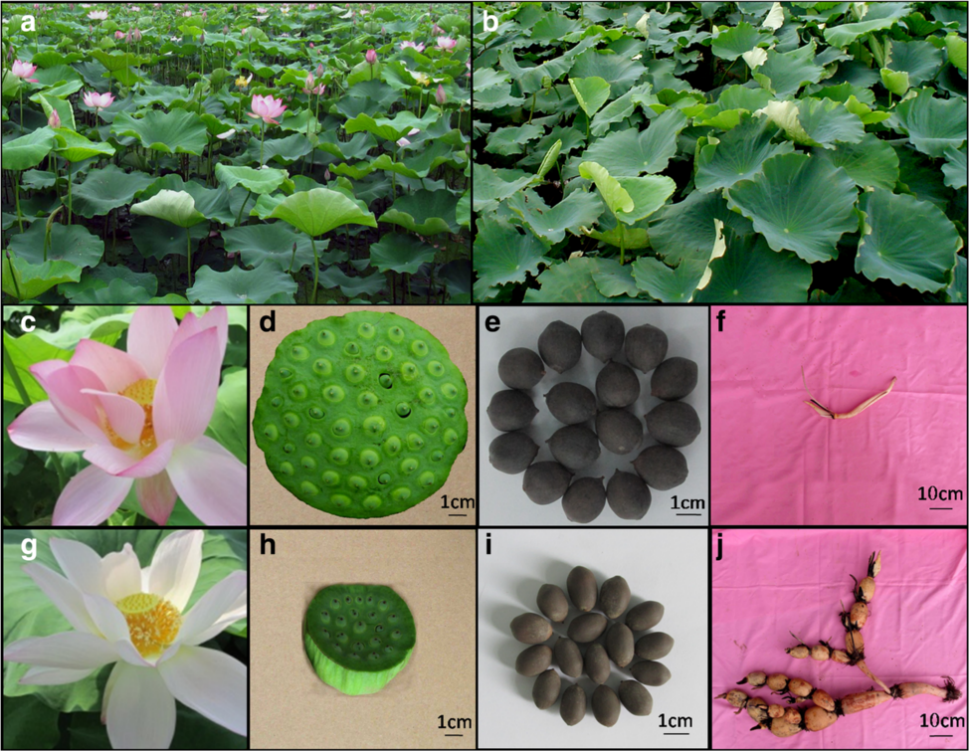
The main phenotypic differences between parent cultivars. **a** the seed lotus cultivar **‘**Mantianxing’ used as female parent was planted in field in fast-growing stage; **b** the rhizome cultivar ‘Juwuba’ used as male parent was planted in field in fast-growing stage; **c**, **d**, **e** and **f** is the flower, fruit, seed and rhizome of ‘Mantianxing’, respectively; **g**, **h**, **i** and **j** is the flower, fruit, seed and rhizome of Juwuba, respectively

Rhizome and seed lotus cultivars display high phenotypic diversity in both vegetative growth and sexual reproduction (Fig. 1). In addition, high genetic diversity has been found between the two types of cultivar [4, 17]. Consequently, a map derived from a cross between a rhizome lotus cultivar and a seed lotus cultivar will substantially facilitate the molecular characterization of phenotypic variation and QTL mapping of important trait loci in both types of lotus cultivar.

In this study, a high-density genetic map was constructed using ddRADseq [16] of genomic DNA from 96 F_2_ progeny derived from a cross between the rhizome lotus cultivar *N. nucifera* cv. ‘Juwuba’ and the seed lotus cultivar *N. nucifera* cv. ‘Mantianxing’. Overall, 71.3 % of the assembled genome of the wild Asian lotus [2] was anchored. This is the first report of a genetic map based on two types of lotus cultivar and will be of value for accelerating the characterization of agronomically important traits.

## Results

### Phenotypic differences between parental cultivars

Although the two cultivars used here, ‘Juwuba’ and ‘Mantianxing’, belong to the same species, they exhibit distinct phenotypic differences. ‘Juwuba’ has vigorous vegetative growth, an enlarged rhizome, and a small number of white flowers; by contrast, ‘Mantianxing’ exhibits vigorous sexual reproduction with a long flowering time and large numbers of red flowers with extra carpels, but its rhizome is smaller (Fig. 1). We have recorded the main characters of both parents for five years (data not show). The rhizome weight was 7.1 fold greater in ‘Juwuba’ than ‘Mantianxing’ and the width of the section between the third and fourth knots of the main stem was 1.54 fold greater in ‘Juwuba’. The numbers of flowers and carpels in ‘Mantianxing’ were 8 and 1.34 fold higher, respectively, than ‘Juwuba’. From the wide phenotypic differences in the parents, we anticipated that broad variation would be present in the F_2_ generation.

### Restriction enzyme selection for DNA sequencing library construction

The assembly sequence of the wild lotus [2] was *in silico* digested with five enzyme combinations EcoRI/SbfI, EcoRI/MspI, EcoRI/SphI, SphI/MluCI, and NlaIII/MluCI. The distribution of restriction fragments on the genome was predicted. As shown in Additional file 1, the MspI/EcoRI combination was predicted to produce 157,936 restriction fragments of 250 to 500 bp; this was the maximum predicted fragment number of the five enzyme combinations, and was considered sufficient to construct a high-density genetic map. Therefore, we double digested the genomic DNA of parents and progeny with MspI and EcoRI to construct a sequencing library.

### DNA sequencing

**Fig. 2:**
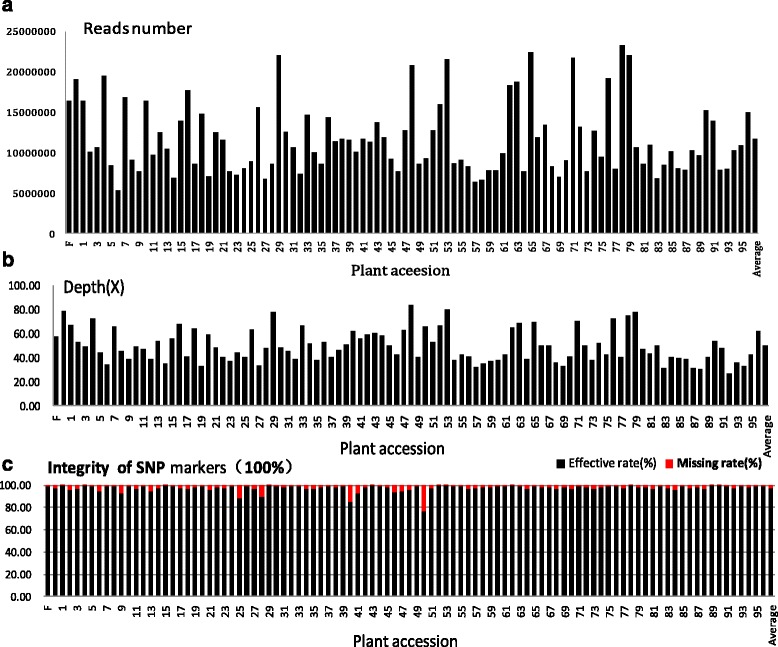
The reads number (**a**), read coverage (**b**) and integrity of SNP markers (**c**) of parents and 97 F2 progenies. Depth: mapped reads depth on the genome; integrity of SNP markers: the number of genotyped SNP markers in sample divided by total SNP numbers used to construct genetic map

The ddRADseq library of all samples was analyzed by massively parallel Solexa sequencing, which generated a total of ~133.65 Gb raw data containing 1,088,935,610 pair-end reads of ~100 bp (Table 1). After removing the low-quality reads (>5 bases with Q score >20), approximately 89.44 Gb clean reads were sorted based on identified barcodes. Most of the samples had more than 5 million reads (Fig. 2a, Additional file 2). Sample z35, which only had 1.2 million reads, was omitted from the following analysis.

**Table 1:** Summary of RAD sequencing for SNP calling from the rhizome lotus Juwuba and the seed lotus Mantianxing (*N. nucifera*) and their F2 progenies

Sample	Total reads	Total base (Gb)	Mapped reads	Mapped base (Gb)	Alignment rate (%)	Coverage (%)	Depth (X)
Mantianxing	16,429,464	1.64	14,359,968	1.44	87.40	7.20	57.94
Juwuba	19,032,200	1.90	17,741,926	1.77	93.22	6.56	78.57
Average of F_2_ population	11,136,905	1.38	7,377,795	0.91	65.76	1.46	48.35
Total	1,088,935,610	133.65	730,348,710	89.44	65.00	1.70	48.65

Note: total line, all samples were calculated including parents and F_2_ population

**Table 2:** Summary of eight linkage groups of high-density genetic map

Linkage group	SNP markers	Bin markers	Linkage distance (cM)	Mean distance (cM)	Largest gap (cM)	No. of distorted segregation markers (*P* < 0.05)	No. of SDRs
LG1	3,131	253	188.7	0.75	4.0	22	1
LG2	1,416	100	78.4	0.78	3.9	9	2
LG3	1,155	104	40.2	0.39	4.5	23	4
LG4	682	73	48.5	0.66	2.7	10	2
LG5	716	79	64.5	0.82	4.3	4	1
LG6	760	70	63.3	0.90	8.4	7	1
LG7	719	56	48.9	0.87	3.3	10	2
LG8	392	56	49.0	0.87	2.90	3	0
Total	8,971	791	581.4	0.74	—	88	13

Note: *SDRs,* segregation distorted regions

The sorted reads were aligned to the wild strain lotus genome assembly (792 Mb) [2] using the Burrows-Wheeler Aligner (BWA) program (v0.7.10) [18]. The 17,741,926 mapped reads from the male parent, Juwuba, and the 14,359,968 reads from the female parent, Mantianxing, covered 7.2 and 6.56 % of the lotus genome, respectively. For F_2_ individuals, an average of 7,377,795 reads was mapped to the reference genome [2] with mean coverage of 1.46 % of the lotus genome. The read depth was 57.94× for the female parent, 78.57× for the male parent, and 48.35× for the F_2_ progeny (Fig. 2b). Detailed read information for the parents and the F_2_ individuals is shown in Additional file 2. The raw data of the F_2_ individuals contained many highly redundant reads, which may have resulted from chloroplasts in the leaf tissues that were used for DNA extraction. This contaminating DNA reduced the mapping rate for F_2_ individuals.

### SNP calling and genotyping

The mapped reads of parents and F_2_ individuals were then aligned to the reference genome [2] for SNP calling. Only uniquely mapped reads with one or two mismatches were retained. SNP calling was carried out using the Genome Analysis Toolkit program (v3.1-1) [19] and filtered using a series of stringent selection criteria (detailed in the Methods section). At last, 10,753 valid SNP markers were obtained and the genotyping loci ranged from 8,243 to 10,747 with high integrity. The SNP markers integrity was 99.38 % for ‘Mantianxing’, 98.07 % for ‘Juwuba’, and 97.36 % for the F_2_ population (Fig. 2c).

### Genetic map construction

The 10,753 SNP markers segregating in the F_2_ population were used to construct a genetic linkage map using JoinMap4.1 software [20]. At a LOD threshold of 15, a total of 8,971 SNP markers were clustered into 8 LGs, consistent with the haploid chromosome number of lotus (*n* = 8). The types of the grouped SNP markers in the parents and the F_2_ population are shown in Additional file 3. The SNP markers were next scanned in 100 kb windows on the mapped scaffolds of the reference genome, and co-segregating SNP markers were sorted into bin markers.

In total, 791 bin markers were identified after scanning for co-segregation and were used to construct a genetic linkage map with 8 LGs spanning 581.4 cM and an average marker interval of 0.74 cM (Table 2, Additional file 4). Detailed information on the SNPs and the bin markers on the linkage map are shown in Additional file 5. LG sizes varied widely: the largest group, LG1, contained 253 bin markers spanning 188.7 cM, while the shortest, LG8, only included 100 markers over 40.2 cM. The average marker interval of the eight LGs ranged from 0.39 (LG4) to 0.90 cM (LG6) (Table 2).

### Segregation-distorted markers

**Fig. 3:**
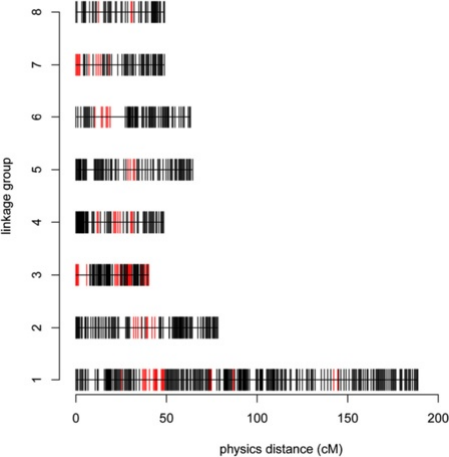
Distribution of normal (black bar) and segregation distorted bin markers (red bar) on 8 linkage groups. The x-axis indicates genetic distance (centiMorgan as unit) and the y-axis represents linkage group number

Distorted segregation (*P* < 0.05) was found for 88 of the mapped bin markers, i.e., 11.1 % of the total (Table 2). The majority of the markers showing segregation distortion were distributed as clusters; in line with previous studies, we defined clusters of more than three adjacent loci showing significant segregation distortion as segregation distortion regions (SDRs) [10, 11]. Thirteen SDRs distributed across seven linkage groups were identified (all LGs except LG8), with the largest number (4 SDRs) on LG3 (Fig. 3; Table 2). These segregation distortion markers may result from gametic or zygotic selection [12], but their presence does not have a large effect on further use of the map for QTL mapping [13–15]. Therefore, the segregation distortion markers were retained here to increase the coverage of the linkage groups.

### Genome scaffolds anchoring of the wild strain of sacred lotus

In our previous work, we partially sequenced the genome of the wild Asian lotus and generated a construct of 792 Mb that accounted for 85 to 95 % of the estimated complete lotus genome [1]. Here, we anchored 809 scaffolds to eight lotus pseudochromosomes with a total length of 565 Mb, representing 71.3 % of the lotus assembly (Table 3); the positions of the scaffolds on the eight pseudochromosomes are detailed in Additional file 5. Of these anchored scaffolds, 164 (20.3 %) could be oriented. The number of anchored scaffolds ranged from 27 for LG8 to 236 for LG1; the physical sizes of the eight pseudochromosomes ranged from 177.95 Mb (LG1) to 20.90 Mb (LG8). The relationship between genetic distance (in cM) and physical size (in Mb) varied widely, from 0.52 cM/Mb on LG3 to 2.34 cM/Mb on LG3, with an average of 1.03 cM/Mb.

**Table 3:** The scaffold number, total length, physical size, marker density and effective length of eight pseudochromosomes

Pseudochromosome	No. of Scaffold	Total length (bp)	Linkage distance (cM)	Mb/cM	Effective length (bp)
chr1	236	177,951,281	188.7	1.06	173,848,305
chr2	149	90,702,750	78.4	0.86	88,660,738
chr3	129	77,900,568	40.2	0.52	76,335,923
chr4	67	48,236,206	48.5	1.01	47,320,720
chr5	74	52,360,038	64.5	1.23	51,365,207
chr6	77	49,297,711	63.3	1.28	48,249,537
chr7	50	48,522,756	48.9	1.01	47,495,387
chr8	27	20,903,902	49.0	2.34	20,529,685
Total	809	565,875,212	581.4	1.03	553,805,502

Note: total length, pseudochromosome length containing unknown base pairs in genome assembly; effective length, the pseudochromosome length without unknown base pairs

### Comparative genomics

**Fig. 4:**
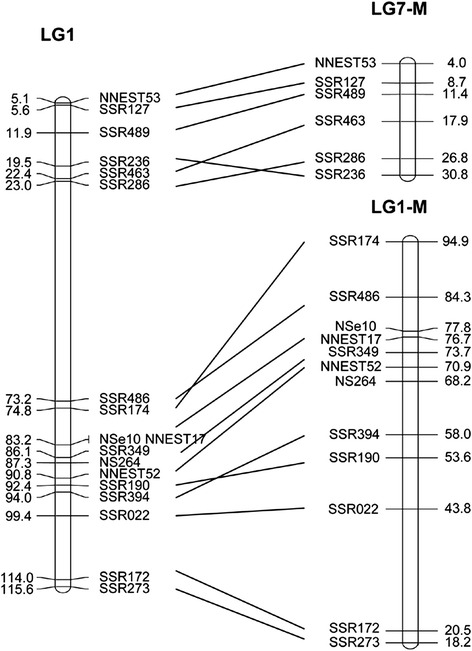
Comparison of LG1 in this study with LG1-M and LG7-M in SSR-based maps [9]. SSR marker information came from Yang et al. [9]

Next, we compared our newly generated genetic map with the two published SSR-based maps [9]. We analyzed 137 SSR markers from the *N. lutea* cultivar ‘AL1’ and 36 SSR markers from the *N.nucifera* cultivar ‘China Antique’. Results showed that 111 and 30 markers respectively were shared with the wild lotus genome [9], and 58 and 19 markers, respectively, could be mapped to the anchored scaffolds of our new genetic map. The correspondence between genetic maps is shown in Table 4. Most, if not all, markers on LG1-M and LG7-M (LG-M = linkage group of male parent) from *N.lutea* were assigned to LG1 in our genetic map (Fig. 4). In agreement with Zhang et al., who integrated LG4-M and LG1-M into LG1 of the RADseq marker-based group [10], we infer from our results that LG1-M, LG4-M, and LG7-M all belong to LG1 in our map. The *N. lutea* markers on LG2-M predominantly fell into LG4, LG3-M into LG7, LG5-M into LG5, LG6-M into LG6, LG8-M into LG3, LG9-M into LG2, and LG10-M into LG8 of our genetic map.

**Table 4:** The correspondence between our genetic map and two published SSR-based maps [9] for scoring SSR markers

Mantianxing × Juwuba	Male parent linkage group (number of SSR markers)							Female parent linkage group (number of SSR markers)									
	1	2	3	4	5	6	7	1	2	3	4	5	6	7	8	9	10
LG1	0	4	1	0	3	0	2	12	1	2	2	0	0	6	0	0	0
LG2	0	0	0	0	0	0	0	0	0	0	0	0	0	0	0	2	0
LG3	1	0	1	0	0	0	0	1	0	0	0	0	0	1	5	0	0
LG4	0	0	1	0	0	0	0	0	8	0	0	0	1	0	0	0	0
LG5	0	0	0	0	0	0	0	0	0	0	0	1	0	0	0	0	0
LG6	2	0	0	1	0	0	0	0	0	0	0	0	5	0	0	0	0
LG7	2	0	0	0	0	0	0	0	1	6	1	0	1	0	0	0	0
LG8	0	0	0	0	0	1	0	0	0	0	0	0	0	0	0	0	1

Note: numbers in the table are shared SSR markers between Mantianxing × Juwuba map and the lotus genetic map constructed by Yang et al. ( the male and female genetic map) [9]

We also compared our genetic map with a high density American lotus map [10]. We found that most SSR markers on LG5 from the high density American lotus map fell into LG1 in our map. Considering that the LG1s from the two genetic maps analyzed above belong to the same chromosome, we can further integrate LG1 and LG5 from the American lotus into one linkage group.

## Discussion

Molecular genetic markers provide a powerful tool for associating heritable traits with underlying genetic variation. However, due to technical limitations, most of the initial large-scale genetic marker development and high density genetic map construction was carried out in model species, such as *Arabidopsis* [21] and rice [22]. In recent years, the development of NGS technology, especially ddRADseq, has provided a powerful and low-cost tool for the large-scale discovery of SNP markers in any plant species [15, 23]. This approach has been successfully applied to the construction of high-density genetic maps [24, 25], assembly of genome scaffolds [26–28], and mapping of QTLs [29, 30].

In the present study, we successfully constructed a high-density genetic map from two *N.nucifera* cultivars using the ddRADseq method. We identified 791 bin markers distributed across 8 LGs; the number of LGs exactly matched the number of chromosomes in *Nelumbo* species (*n* = 8). The average interval between genetic bin markers in this study is 0.74 cM. This is comparable with or better than the marker density of genetic maps constructed for other crops using the same RADseq protocol, e.g.,sesame,1,272 markers and 1.2 cM interval [31], soybean, 5,785 markers and 0.43 cM interval [32], and peanut, 1,685 markers and 0.9 cM interval [33]. In addition, our new genetic map provides a marked improvement in marker density and population size compared to the two previously published genetic maps for *N.nucifera* and *N.lutea* [10], which were based on the same F_1_ population of 51 individuals. The published map for *N. nucifera* contains 224 markers, and that of *N. lutea* has 3,895 RADseq markers and 156 SSR markers on 9 LGs. The genetic map produced in the current study is therefore a more saturated map and has high resolution for QTL mapping.

Using the 8,971 ddRAD markers in the present high density genetic map, 809 genome scaffolds of the wild strain *N. nucifera* assembly (from uncultivated sacred lotus) were successfully anchored to eight pseudochromosomes with a total length of 566 Mb (71.3 % of the lotus genome). The previously published scaffold assembly for lotus consists of nine megascaffolds indicating that a gap was present within a linkage group of one chromosome. The additional anchoring in this study resulted in a match between the number of linkage groups and the haploid number of chromosomes; moreover, the relative sizes of the linkage groups are similar to those of the chromosomes [34]. Therefore, our new genetic map, along with the improved genome assembly, resolution, and integrity, will play an important role in further marker assisted selection and QTL mapping of important traits for breeding new varieties of lotus.

Segregation distorting is a common phenomenon in most genetically segregating populations [35–37] and is thought to be an important driving force in evolution through increasing genomic heterozygosity. We also observed a low rate of segregation distortion affecting some markers in our genetic map. The rate of such markers was less than one-third that reported for the SSR based American map [8]. Two factors may account for this difference. Firstly, our map used an F_2_ population whereas the American map was based on an F_1_ population. In general, segregation marker skewing is lower in F_2_ populations than in recombinant inbred lines, backcross populations, and F_1_ populations [38]. Secondly, the two *Nelumbo* species used here as parents show less genetic divergence than is present between the American lotus and the Asian lotus. Mapping based on wide crosses carries the risk of distorted segregation and altered linkages due to gametic selection and/or chromosomal rearrangements [35]. We further found that most of the aberrant segregation markers tended to be located in close proximity and 11 putative SDRs were detected; this indicated that segregation distortion was not induced by technical problems but most likely arose through biological factors such as selection for gametophytes and sporophytes.

Comparison of genetic maps for *N.nucifera* and *N. lutea* showed good collinearity in all the linkage groups (Fig. 4). Markers on LG1-M and LG7-M of *N. lutea* generally appeared in the same order as those from LG1 of *N.nucifera*, although a few adjacent markers showed a reversal in their order. The discrepancies in the order of adjacent markers might be due to the relatively small populations used for linkage map construction (51 individuals for the genetic map of *N. lutea* vs. 96 individuals for *N.nucifera*); however, we cannot rule out the possibility of genetic variation between the two species. The long genetic length and high density of markers in our genetic map provided the opportunity to fill gaps in previous maps of *N.lutea*. We successfully integrated LG1-M, LG4-M, and LG7-M into a single linkage group (LG1). Moreover, we integrated LG1 and LG5 from the RADseq map [10] into a single linkage group, which further saturated the high density map for American lotus.

Although 96 F_2_ progeny were used to construct the genetic map and 8972 SNP markers (0.064 cM per SNP marker) were obtained, only 791 bin markers were identified due to co-segregation in the F_2_ population. The small genetic pool resulted in lower recombination in the F_2_ individuals and reduced the resolution of the genetic map [27]. Currently, we are using asexual reproduction of F_1_ progeny to increase the number of F_2_ progeny to over 300. Using this expanded resource, we will soon obtain more SNP markers to further saturate our genetic map. As the F_2_ population can be preserved permanently through asexual reproduction, this will facilitate the characterization of important traits in the laboratory and field.

## Conclusions

In this paper, we use cutting-edge genomic techniques to create genetic map of *N. nucifera* that greatly improves on the quality of previous genetic maps. *N. nucifera* is valued as a food crop in addition to its ornamental and cultural importance, yet no genetic map that we are aware of focuses on agricultural cultivars. We have filled that gap. In addition, because of the higher density and quality of information in this genetic map, we are able to anchor the “scaffold” portions of the recorded *N. nucifera* genome assembly that are not based on continuous, contiguous sequences. With a better genome assembly, the genotypic basis of valuable traits can be identified, and sacred lotus breeding can be practiced more efficiently.

## Methods

### Plant breeding and DNA extraction

Two cultivars of *N. nucifera* were used in this study: ‘Juwuba’, a high-rhizome-yield cultivar, and ‘Mantianxing’, a high-seed-yield cultivar. They are the most popular rhizome and seed lotus cultivars grown in the Yangtze valley. These two accessions were developed and preserved by National Germplasm Wuhan Aquatic Vegetable Garden, Institute of Vegetable, Wuhan Academy of Agriculture Science and Technology and can be open accessed (ATTEN: Weidong Ke, email: wdke63@163.com). Lotuses are not endangered or protected species in China and lotus research and field studies were authorized by local government. The parental generations and F_2_ plants were planted and preserved through asexual reproduction in six-m^2^ containers individually at the National Germplasm Wuhan Aquatic Vegetable Garden in Wuhan city. Key morphological and agronomical characteristics of both parents were analyzed.

For DNA extraction, young leaves of the parental plants were harvested from the underground stem tip developed from the rhizome; for F_2_ individuals, the primary leaves from germinating seeds were used as young leaves on underground stem tips were too short. Approximately 0.3 g young leaves per sample were collected, treated with liquid nitrogen and grounded into powder. Genomic DNA was then extracted using the cetyltrimethyl ammonium bromide (CTAB) method [39] with the minor modification that 4 % PVP 40 was added to the CTAB buffer.

### In silico analysis of restriction enzyme-recognition sites on the reference lotus genome

The sequence of the wild strain lotus genome assembly [2] was used to identify the best restriction enzyme combination for sequencing library construction. The assembly is 792 Mb in length and includes 3031 scaffolds (>2 kb); scaffold N50 was 986.5 kb (a long scaffold N50 generally indicates a high-quality assembly). In silico double-restriction digestion of the lotus genome with five enzyme combinations was carried out using Perl script developed by ourselves (https://github.com/Nextomics/ddRAD-pipeline). The tested enzyme combinations were EcoRI/SbfI, EcoRI/SphI, SphI/MluCI, EcoRI/MspI and NlaIII/MluCI. This in silico procedure provided the distribution of estimated digestion sites and of resultant fragment lengths.

### Genotyping

The restriction enzymes MspI (5ʹC/CGG3ʹ) and EcoRI (5ʹG/AATTC3ʹ) were selected for constructing ddRADseq libraries; the modified method described by Peterson et al. [16] was used. After digestion of the genomic DNA at 37 °C, DNA fragments were purified and ligated to MspI and EcoRI adapters. The MspI adapter contains a 4 to 6 nucleotide barcode for sample recognition. DNA fragments between 300 and 500 bp were separated and enriched by PCR amplification. The PCR products were gel-purified and used for pair-end sequencing on the Illumina High-seq2500 sequencing platform (Illumina, Inc.; San Diego, CA, U.S.), following standard protocols.

Based on the Illumina raw data, a custom Perl script was used to sort sequences of individual samples based on indices and trimmed barcode sequences for faster processing. Only sequences containing the barcode followed by an EcoRI or MspI recognition site were retained. Low-quality, contaminant sequences were further filtered using the NGS QC Toolkit [40] and those with more than three missing nucleotides were deleted. The clean data was then mapped to the wild lotus draft genome [1] using the BWA program [18]. Sequences that contained more than two mismatches and multi-mapping reads were excluded. The remaining high quality reads were used for SNP calling using Unified Genotyper in the Genome Analysis Toolkit v3.1-1 (GATK) [19]. The SNPs were filtered as follows: cluster window size 10, quality of depth (QD) <3 and genotype quality (GQ) <20. For each given SNP site, the genotypes were labeled as “A” for a homozygous genotype of reference alleles, “B” for homozygous genotype of alternate non-reference alleles, and “H” for the heterozygous genotype. The low quality (QD <3) and clustered SNP sites were removed from the genotype data, and the genotypes occupying lower reliability (GQ <20) were replaced by a missing value.

### Genetic map construction and anchoring the lotus wild strain genome assembly

SNP calling was carried out using GATK (v3.1-1) [19] and filtered at the population level according to the following criteria: sequencing depth of SNP sites between 40 and 10,000; mutation rate at each SNP site of less than 5 %; and, fewer than 10 % missing SNP sites. SNPs that were heterozygous in both parents were also excluded. The remaining SNP markers were used to construct a linkage map using JoinMap4.1 software [20]. Initial linkage groups (LGs) were established at a LOD threshold of 5 to 20. Using a LOD threshold of 15, most markers could be assigned to 8 LGs. After the markers were grouped, a draft genetic map was constructed. The data points where the genotype data were in disagreement with both flanking data were defined as “singletons.” Along the marker order, the markers with more than five “singletons” were excluded using Microsoft Excel, and then the remaining markers were reordered. After grouping, an in-house Perl script (https://github.com/Nextomics/ddRAD-pipeline) was used to trim marker orders by moving the markers in the same scaffold, and to determine marker orientation in linkage group. The SNP markers were further scanned in 100 kb windows on the mapped scaffolds of the reference genome, and then renamed bins were created to represent continuous co-segregation markers. This process was repeated until deleting markers did not affect marker order. Markers orientation was determined by the trend of linkage distance. Then a new genetic map was constructed and the Kosambi function was used to convert recombination frequencies to relative distances in centimorgans (cM).

After the bin markers had been ordered on the genetic map, the position of the scaffold was then anchored on the basis of bin marker order. Scaffolds containing two or more bin markers were further oriented based on the order of the bin markers on the genetic map and their position on the scaffold.

### Linkage map comparison

The constructed genetic map was compared with maps of *N. nucifera* ‘China Antique’ and *N. lutea* ‘AL1’. Primers for the SSR markers were first aligned to the reference genome with Bowtie software (v2.2.3) [41], then aligned SSR markers with short repeat sequence domains were used to further anchor the scaffolds of the constructed genetic map and marker positions on the genetic map were confirmed.

## Abbreviations

BWA, Burrows-Wheeler Aligner; cM, centimorgan; CTAB, cetyltrimethyl ammonium bromide; ddRADseq, double digest restriction site-associated DNA sequencing; GATK, Genome Analysis Toolkit; GQ, genotype quality; LG, linkage group; LOD, “logarithm of the probability ratio”, a well-established linkage analysis; MAS, marker-assisted selection; NGS, Next-generation sequencing; PCR, polymerase chain reaction; PVP 40, polyvinylpyrrolidone (N-vinyl pyrrolidone, where *N* = 40); QD, quality of depth; QTL, quantitative trait locus; RADseq, restriction site-associated DNA sequencing; SGR, segregation distortion region; SNP, Single-nucleotide polymorphism; SSR, Simple Sequence Repeat

## Additional files

Additional file 1: Distribution of predicted restriction fragments in silico digested with five enzyme combinations. (TIF 298 kb) Additional file 2: The basic sequencing data information of the parents and F_2_ progenies. (XLSX 25 kb) Additional file 3: Grouped SNPs’ name, position on the scaffold and segregation information in the parents and the F2 individuals. (XLS 13352 kb) Additional file 4: The high-density lotus genetic map consisted with 8 LGs (LG1- LG8, on top of the map). The bin names and locations are labeled on the LGs. (PDF 1069 kb) Additional file 5: The mapped SNP markers’ name, position, type and their flanking sequences and the bin marker information. (XLSX 829 kb)
