# Supplementary figures and images for: Construction of a high-density, high-quality genetic map of cultivated lotus (Nelumbo nucifera) using next-generation sequencing
Source: BMC Genomics. 2016 Jun 17;17:466. doi: 10.1186/s12864-016-2781-4 (PMC4912719; doi:10.1186/s12864-016-2781-4)

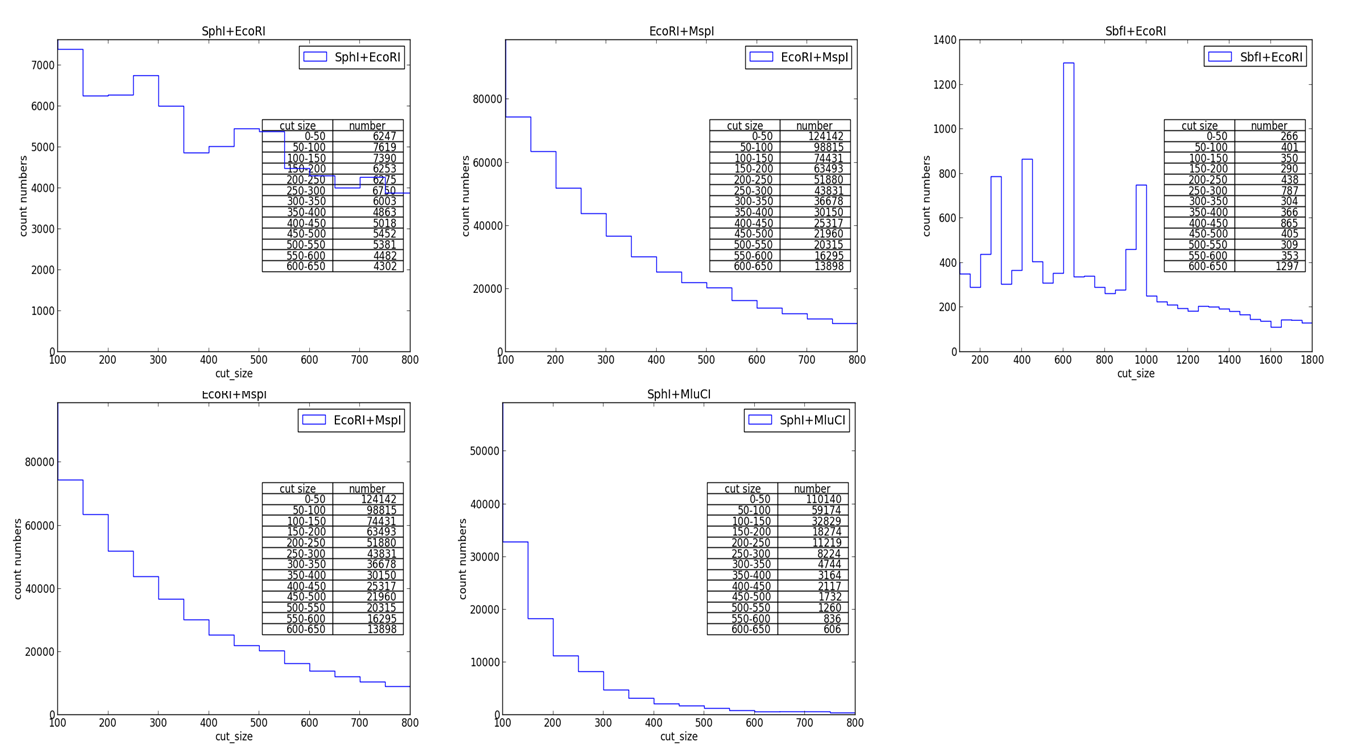

Supplement: Additional file 1: — Distribution of predicted restriction fragments in silico digested with five enzyme combinations. (TIF 298 kb) [file 12864_2016_2781_MOESM1_ESM.tif]

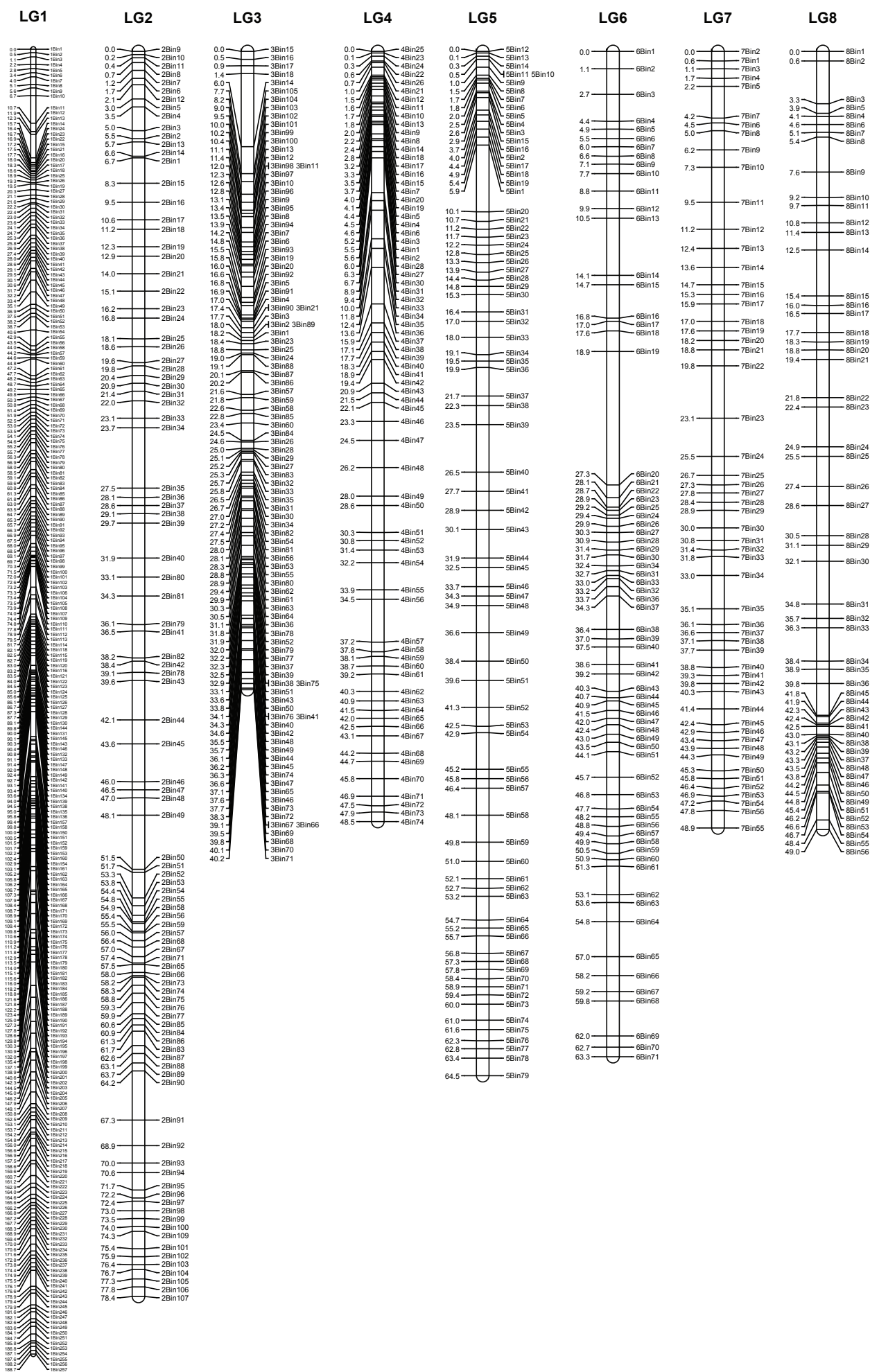

Supplement: Additional file 4: — The high-density lotus genetic map consisted with 8 LGs (LG1- LG8, on top of the map). The bin names and locations are labeled on the LGs. (PDF 1069 kb) [file 12864_2016_2781_MOESM4_ESM.pdf]
